# Principles of engagement on research and other collaborations between the brewing sector and research entities: the FACT Principles

**DOI:** 10.1017/S0007114523000533

**Published:** 2023-10-28

**Authors:** Frans J. Kok, Martin Zarnkow, Aafje Sierksma

**Affiliations:** 1 Emeritus professor Nutrition & Health, Wageningen University & Research, Wageningen, The Netherlands; 2 Research Center Weihenstephan for Brewing and Food Quality, Technical University of Munich, Munich, Germany; 3 The Dutch Beer Institute, Generaal Foulkesweg 72, 6703 BW Wageningen, The Netherlands

**Keywords:** Principles of engagement, Brewing sector, Research entities, Research Collaboration

## Abstract

Public–private partnerships are subject to intense scrutiny. This is specifically the case for sensitive health-related topics such as alcohol consumption. The brewing sector and representatives of the scientific community therefore stressed the need for specific principles for the proper and transparent governance of research and other collaborations between the brewing sector and research entities. At a 1-day seminar, a group of scientists and representatives from the brewing and food sector reached a consensus for such principles. They adhere to the following four fundamental conditions: Freedom of research, Accessibility, Contextualisation and Transparency. The points of focus in the FACT principles are open science, meaning that the methods and results are made accessible and reusable, and relationships are clearly disclosed. Actions to be taken for dissemination and implementation of the FACT Principles are, for instance, publishing them on public websites, including them in formal research agreements, and citing them in scientific publications. Scientific journals and (research) societies are encouraged to support the FACT Principles. In conclusion, the FACT Principles provide a framework for increased transparency and control of funding-related bias in research and other collaborations between the brewing sector and research entities. Monitoring their use and evaluating their impact will help to further refine and enforce the FACT Principles in the future.

The food and beverage industry plays an important role in advancing food science and nutrition. In public perception, research funded by industry and research collaborations between research entities and companies is followed critically. The scientific findings coming from such collaborations are frequently questioned^(1–3)^.

In the food and pharmaceutical industry, principles have been developed to describe how to collaborate with research entities with integrity. Over 25 years ago, the alcoholic beverage industry, together with governments, scientific researchers and the public health community, established principles to cover the main topics of scientific integrity and engagement with scientists in alcohol research^(4)^.

In society, opinions and discussions are intensifying with regards to research funding by industry and collaborations between industry and research entities. In addition, the credibility of scientists with research and advisory relationships with industry is at stake^(5)^. This makes scientists less willing to engage in collaborative activities with the industry, as it exposes them to reputational risk. This is specifically the case for sensitive health-related topics such as alcohol consumption. Excessive consumption of alcoholic beverages of any type can lead to serious psychological and physiological diseases. However, light-to-moderate consumption may have some health benefits. Science helps to find a balance in this double-edged sword of the health effects of alcohol consumption.

The alcoholic beverage industry, including the beer sector, sees it as their social responsibility to investigate and communicate on the health effects of their products. The question is how this can be achieved given the distrust of findings from research funded by the sector and the increasing reluctance of scientists to collaborate with the sector. A good example is the relatively recent Moderate Alcohol and Cardiovascular Health (MACH) trial^(6,7)^ funded by public–private partnership involving NIH’s National Institute on Alcoholism and Alcohol Abuse and the NIH foundation. In theory, this study could have generated highly relevant data on the relation between alcohol and health. The project was however stopped at an early stage for reasons of unclarity about internal NIH procedures and discussions about the study design^(8–12)^. Amongst others, this case stresses the importance of the need, after 25 years^(4)^, for up-to-date specific principles for the proper and transparent governance of research and other collaborations between the brewing sector and research entities, engaging both sides in the importance of independent science. This led the brewing sector and representatives of the scientific community to organise a 1-day seminar. Scientists and representatives of the brewing and food sectors were invited to establish up-to-date principles specific to the brewing sector that pave the way for research entities and the brewing sector to collaborate with transparency and integrity. This article describes how these brewing sector-specific principles were developed and summarises the discussions at the seminar, including recommendations for dissemination and implementation of the principles.

## Methods

### Draft principles

The Brewers of Europe, together with two members of the organising committee (F.J.K./A.S.), drafted the principles. They are mainly based on existing principles for managing conflicts and maintaining trust in alcohol science^(4)^. Furthermore, food and pharmaceutical principles of engagement were also taken into account^(13,14)^.

### Participants

In February and March 2022, sixty experts received an invitation for the seminar. These experts included forty European scientists, twelve representatives of the brewing sector and eight representatives of the food sector.

The scientists were selected based on their expertise, working in the field of brewing, nutrition, health and medical, or psychosocial sciences. Invitees needed to be familiar with collaboration with the industry, preferably in the alcohol sector. The European Foundation of Alcohol Research (ERAB) database was used to select the scientists^(15)^. For 13 years, ERAB funded biomedical and psychosocial research into beer and other alcoholic drinks, focussing on positive as well as negative aspects. In addition, several scientists who presented at Beer and Health Symposia^(16)^, organised by the Beer and Health Initiative, were invited.

Representatives of the brewing sector were employees of The Brewers of Europe as well as some of their members familiar with collaborating with research entities. Representatives of other food sectors were invited to learn from their experiences in collaborations with research entities.

Some could not accept the invitation because of other obligations or indicated that they had no interest in participating, and a few did not reply after several reminders. Four of the invited scientists rejected participation because the seminar was funded by the brewing sector.

In total, twenty scientists, eleven representatives of the brewing sector and three representatives of the food sector accepted the invitation (see overview of participants at the end of the paper). One month before the seminar, they received the draft principles with the request to give their initial comments/suggestions, which were included in the principles that were discussed on the seminar day.

### Seminar day

The seminar was organised on June 24, 2022, at the Research Center Weihenstephan for Brewing and Food Quality, Technical University of Munich, Germany. One representative of the brewing sector could not attend because of unexpected obligations, and one representative of the food sector could not attend due to illness.

The seminar was structured to explore the relevant issues, allowing detailed discussions with the final aim of reaching a consensus on the principles. To allow the participants to speak freely and without restrictions at the seminar, the discussions described in this paper do not reveal, either explicitly or implicitly, who said what.

On the morning of the seminar day, the principles were presented one by one by the chair (FJK) and discussed in a plenary session. The participants were free to provide any inputs. This could range from changing or adding of wordings to improve comprehensibility, to further elaborating on certain topics, e.g., to give a balanced view or making the principles better workable in practice. Three participants volunteered to be editors. They took note of the comments expressed during the plenary session on the principles and proposed revised principles that were presented to all participants in the afternoon. Finally, participants discussed how the principles could be disseminated and implemented in practice.

Right after the seminar, the principles were shared with the participants for a final check and approval. At this stage, the input, if any, was mainly textual.

## Results

### The FACT principles

### Principles of Engagement on research and other collaborations between the brewing sector and research entities

#### Preamble

Beer is a fermented beverage (regularly 0–12 % alcohol by volume) that has been consumed worldwide for thousands of years. It is a legitimate topic of scientific interest. The brewing sector has a long history of cooperation with research entities, such as universities, research centers and laboratories, either public or private. Research has focussed on the ingredients and properties of beer; the brewing process; and the nutritional, physiological and psychosocial aspects of beer consumption and its effects on human health. Alcohol consumption can lead to serious psychological and physiological diseases depending on alcohol content, drinking frequency and pre-existing health conditions, among other things^(17)^. And there are major social harms that can be caused by alcohol abuse, e.g., antisocial behaviour and unsafe sex^(18)^. However, light-to-moderate consumption of alcoholic beverages of any kind is associated with some health-promoting effects^(19)^. Science helps to find a balance in this double-edged sword of the health effects of alcohol consumption.

Research projects funded by industry and research collaborations between research entities and companies are being viewed increasingly critically. The results of such research are often questioned regardless of scientific quality. This also applies to cooperation between the brewing industry and research entities. This critical view is recognised by the brewing sector. The social and health relevance of alcohol consumption requires a rigorous and transparent approach to research on these issues. In collaboration between the brewing sector and research entities, it is of utmost importance to establish transparent and clear guidelines and to ensure these are followed.

A group of scientists and representatives of the brewing and food sectors have jointly set themselves the task of defining brewing sector-specific, transparent principles for research cooperation that recognise, prevent or exclude conflicts of interest and enable independent research to the highest scientific standards. The principles, as presented below, were developed by this group of scientists and representatives of the brewing and food sectors at a 1-day seminar, and they adhere to the following four elemental conditions: Freedom of research, Accessibility, Contextualisation and Transparency. Attendees at the seminar recommended that these so-called FACT Principles should form the basis for the proper and transparent governance of research and other collaborations between the brewing sector and research entities. They are based on the Dublin Principles^(4)^ and food and pharmaceutical principles of engagement.

Both the brewing sector and research entities must comply with the highest professional, scientific and ethical standards in conducting and reporting beer research, regardless of the source of funding. All research must adhere to the fundamental principles of research integrity as defined by the European Code of Conduct for Research Integrity^(20)^ as well as the Standard Operating Procedures for Research Integrity^(21)^.

The FACT Principles:

- Apply to all research collaborations between the brewing sector and public or private research entities, covering any research method and question. The FACT Principles do not apply to research that cannot commit to full transparency because of competitiveness and intellectual property rights.
- Apply to any other collaborations between the brewing sector and research entities, such as a scientist speaking for the brewing sector or at a symposium organised by the brewing sector, membership of a (scientific) committee for the brewing sector or providing scientific advice to the brewing sector.

#### Principle 1: freedom of research

The brewing sector and research entities should be able to investigate, independently or in collaboration, the whole spectrum of beer and beer consumption, with the purpose of gaining a better understanding of, for instance:

- The ingredients and properties of beer,
- The brewing process,
- The relationships between beer and all negative and positive nutritional, societal and cultural outcomes, including health and disease.

#### Principle 2: transparency

Research collaborations as well as any other type of collaboration between the brewing sector and research entities should be made transparent:

- In research collaborations, both parties are encouraged to actively endorse Open Science rules of conduct, meaning that methods and results are made accessible and reusable by all interested parties in society.
- Those involved in research or in communicating study results should adhere to the general principles of disclosing any interest that might be perceived as potentially affecting the design, conduct, analysis, interpretation or reporting of the research project. Moreover, all types of financial and/or in-kind support for research activities need to be acknowledged in any dissemination of the research.
- Any other communication by the brewing sector or by research entities collaborating with the brewing sector for research into beer or including beer (not specifically research funded by the brewing sector) should disclose the sender as well as any interests of the sender.

#### Principle 3: accessibility

As part of Open Science (Principle 2a), research conducted in collaboration between the brewing sector and research entities should be freely accessible. Both parties have a responsibility and should agree, irrespective of the outcomes, to:

- Making the research data available in public repositories following existing standards such as the FAIR Principles.*
- Publishing all the results of their work in independent peer-reviewed journals, preferably open access journals, or otherwise disseminate their findings, for example, in publicly available reports or documents.

The collaborating parties should agree in advance on the planning of the scientific publication and further communications about the research project. In addition, they should agree under which circumstances, if any, publication can be delayed and for how long. Reasons may include the time needed to protect ethics and data safety, the time needed to protect proprietary information or to prepare for a public response in case of an unexpected outcome. Any delayed agreement in publication and communication will be overruled when the outcomes have whole-of-society public-health implications.

#### Principle 4: contextualisation

The communication of study results from research collaborations between the brewing sector and research entities or from research into beer or including beer in general (not specifically funded by the brewing sector) must comply with all relevant legislation, such as the EU or national legislation. For a better understanding of the research and to present a balanced picture, the results should be placed in the context of evaluated and recognised research and should consider the totality of evidence.

## Discussion

Collaboration between industries and research entities is important for knowledge generation and for scientific advancement and to address societal issues. However, public–private partnerships are subject to intense scrutiny. The FACT Principles described in this paper provide guidance for a mutually acceptable means of collaboration, both in research and in any other collaboration between the brewing sector and research entities that has the potential for societal benefit. The central concepts of the principles are open science, which means that the methods and results are made accessible and reusable, and relationships are clearly disclosed. This is in line with the recently updated framework for minimising bias and promoting integrity in industry-funded research by the Institute for the Advancement of Food and Nutrition Sciences, which also focuses on increased transparency and open science^(23)^.

### Limitations and strengths

The FACT Principles should be considered in light of their limitations and strengths. First, much of the criticism of industry-sponsored research is related to the idea that the funder interferes with the choice of research topic, research methods, or interpretation of the research findings. It is hard to completely dispel this criticism. There are many ways of collaboration possible between industry and academia. As in any good collaboration, it should be possible to discuss the research question and setup with all involved. Often knowledgeable scientists are employed in the private sector^(24)^ who can also provide valuable input. There should be transparency about this, and it is already common practice in scientific articles to describe the responsibilities of the authors and the role of the funder. The shift towards Open Science, as described in the FACT Principles, allows peers to review and provide feedback on the research design itself before the study is initiated. As such, this can help to eliminate questionable research practices.

Second, these principles were set up by a group of thirty-two experts, with the aim of representing the majority view of all those concerned with research and other collaborations between the brewing sector and research entities. The invited scientists might be seen as not representative, as they were selected based on their familiarity with collaborations with the industry. As such they may be considered to have potential conflicts. However, because they have worked in public-private partnerships, they are aware of the sensitive topics that need to be expressed in the principles. The latter might be difficult to capture for those who do not have links with the brewing sector or other industry. They might have the merit of being independent, but the disadvantage that they may not understand, or engage with, the complexity of the issue. During the process of establishment of the FACT principles, the attendees were given ample opportunities to engage with the principles; before the seminar, they could provide input; during the seminar, there were lively discussions on the principles and after the seminar, all participants were asked to comment on or approve on the principles. Seminar participants agreed that what is presented in this article reflects the discussion and expands upon the seminar. Because these principles have not yet been implemented, the FACT Principles should be seen as a living document that might require refinements in the future to be applicable in practice and to reflect ongoing discussions.

Finally, having well-defined principles does not guarantee that any research or collaboration is seen without conflicts of interest by the outside world. There will always be advocates in society who support isolating the (brewing) industry from any participation in research or who are sceptical about any research funded by the (brewing) industry. Some scientists will remain reluctant to engage with the industry, but there are also scientists who feel that collaboration with industry can be valuable for knowledge generation and help solve major societal problems. The seminar participants agreed that it is good to develop principles of engagements in collaborations between the brewing sector and research entities and its subsequent acceptance and adherence by both parties as a basis for transparent cooperation, while respecting competition law.

### Commentary on the preamble

An important point that came up at the seminar is that the Preamble should reference that alcohol is a very delicate and controversial topic in the field of medical research. On the one hand, alcohol consumption is associated with a series of diseases, including serious clinical conditions such as liver cirrhosis, some cancers and cardiovascular diseases (CVD)^(17)^. In addition, alcohol abuse can cause major social harms such as violence and antisocial behaviour, unsafe sex, accidents and injury^(18)^. On the other hand, light to moderate consumption may be associated with health-promoting effects, such as a reduced risk of CVD and type 2 diabetes mellitus, reducing the overall mortality in the age groups at risk^(19)^. Both aspects depend on factors such as the alcohol content, drinking frequency and pre-existing health conditions. Science helps to find a balance in this double-edged sword of the possible health effects of alcohol consumption.

The FACT Principles, while having near universal utility, do not apply to research that cannot commit to full transparency because of competitiveness and intellectual property rights. Representatives of the brewing sector indicated that this is, for example, the case for research focussing on the ingredients, process and quality of the products and research that is related to new product development.

### Commentary on principle 1: freedom of research

The principles should be applicable to all research and communications, including both the negative and positive aspects. Therefore, it was decided to specifically stress this in Principle 1 (Freedom of research) in the last bullet as follows: ‘The relationships of beer with all negative and positive nutritional, societal and cultural outcomes, including health and disease.’

### Commentary on principle 2: transparency

Seminar participants generally recognised that transparency, as described in Principle 2, is a major step to implement for the private sector as well as for research entities collaborating with the private sector. This requires extra work and effort preceding any study: preregistering the research question and hypotheses, preregistering the design and study setup, as well as an analysis plan. This allows peers to review and provide feedback on the research design before the study is initiated. As such, this can help eliminate questionable research practices. After data collection, transparency means uploading all data and study materials to publicly available repositories, as well as publishing in open-access journals by way of preference.

Regarding Open Science, Principle 2a states: ‘… both parties are encouraged to actively endorse Open Science rules of conduct…’. Although the wording ‘encouraged’ indicates that it is not a must, the aim in the end is, of course, to strive to adopt as many practices and resolutions as possible in order to engage in Open Science. During the seminar, it was specifically highlighted that one should be aware that the data are uploaded to publicly available repositories (as also stated in Principle 3a) and not sold to (closed) repositories. Open Science Framework^(25)^ is an example of a suitable platform for implementing open science.

Transparency on the conflict of interest is an important factor in increasing public trust in science. Principle 2b covers this by stating ‘…of disclosing any interest that might be perceived as potentially affecting the design, conduct, analysis, interpretation or reporting of the research project. …’. It is not always clear which interests are relevant to declare. Efforts to standardise disclosure guidelines have, for example, been made by the International Committee of Medical Journal Editors^(26)^ and the American Society for Nutrition Model Disclosure Form of the American Society of Nutrition^(27)^. These forms can serve as a model for disclosing any interest in research or other collaborations between the brewing sector and research entities. In case of doubt, it is better to declare than discover afterwards when being questioned that it was not declared.

Some general remarks on the methodology of the research were provided when discussing Principle 2. First, it must be ensured that the optimal and most advanced methodologies are applied to the hypotheses being generated or tested. Special attention should be paid to valid reference groups in observational studies and control groups in intervention studies. Where relevant, good clinical practice and good laboratory practice should be applied. Good clinical practice is applied to drug trials and is often stricter than needed and not always feasible for studies in nutritional sciences. Although there is currently no standard code for good nutritional practice, there are ongoing discussions about the development of such a standard^(28)^. In the short term, a document will be published on adaptations to the CONSORT principles for research into nutrition. Furthermore, guidelines have recently been drafted to make press releases on results of nutritional studies and how to review a press release on nutritional studies^(29)^. Second, transparency also means that the limitations of the research and consequences for the validity and generalisability of the results need to be described.

### Commentary on principle 3: accessibility

Principle 3 highlights the importance of attempting to publish findings from all research in, preferably, open-access peer-reviewed journals. This must be done regardless of whether the results are unanticipated or null, and also when multiple submissions are required. If it is not possible to publish (for any reason) in a scientific journal, the data should be made publicly available in reports or documents, for example in Open Science repositories.

The last paragraph of Principle 3 stresses that ‘in order to avoid ethical and data safety issues and to protect proprietary information, publication and dissemination may be subject to reasonable and ethical restrictions agreed in advance’. This may result in a delay in the accessibility of data. However, such restrictions can be overruled when the outcomes have direct consequences for individuals or broader public-health implications. Examples are serious adverse events in which the outcome is death, life-threatening situation, hospitalisation (initial or prolonged), disability or permanent damage, congenital anomaly/birth defect or interventions are needed to prevent permanent impairment or damage^(30)^.

### Commentary on principle 4: contextualisation

In Principle 4, it says: ‘For a better understanding of the research and to present a balanced picture, the results should be placed in the context of evaluated and recognised research and totality of evidence’. This means that statements on the increased and reduced risk must be placed in the context of absolute lifetime risk, to make the scale of the issue clear.

Providing a balanced overview of other evidence on the research topic is essential to understand the impact of the new research findings. This could range from new findings in a specific area, contradictory findings, or findings that confirm earlier demonstrated relationships.

### Dissemination and implementation of the FACT principles

After publishing these FACT Principles, they should be disseminated and implemented. During the seminar, the following suggestions for action were made:

- The Brewers of Europe, as an overarching organisation for the brewing sector in Europe, should publish the principles on their website and actively recommend the principles to their members. Monitoring should take place and cover to whom the principles have been shared, whether they are being used, and (how) they work out in practice. To increase the dissemination and implementation of the principles in the brewing sector, it was suggested that training should be provided about the FACT Principles to brewing sector employees who are engaged in research, or commissioning research. Ideally, these principles should be included in calls for research proposals and formal research agreements.
- Research entities can be involved in the dissemination of the FACT Principles in different ways. This should be accomplished by sharing the principles with scientists who are or have been working with the brewing sector. Relevant (research) societies as well as universities should be actively approached, informed and asked to support the principles. In addition, editors of relevant scientific journals need to be approached with arguments to include the FACT Principles in their author guidelines. Monitoring is required to evaluate the impact of these actions.
- When research or any other collaboration is conducted according to the principles, this should be cited (with reference to this paper) in the publication. Counting the citations of this paper in scientific publications or in other (social) media (e.g., by using Altmetric or Plumx) can help to monitor to which extent the principles are adopted and implemented.

### Conclusion

The FACT Principles described in this article provide a framework for increased transparency and control of funding-related bias in research and other collaborations between the brewing sector and research entities. The points of focus in the FACT principles are open science, which means that the methods and results are made accessible and reusable, and relationships are clearly disclosed. It is recommended that the principles set out in this paper are accepted and implemented by the brewing sector, other scientists, academic institutions, relevant (research) societies and scientific journals. Monitoring their use and evaluating their impact will help to further refine and enforce the FACT Principles in the future.

## Footnotes

* The FAIR Guiding Principles for Scientific Data Management and Stewardship provide guidelines to improve the Findability, Accessibility, Interoperability and Reusability of digital assets^(22)^.
