# Principles of engagement on research and other collaborations between the brewing sector and research entities: the FACT Principles – CORRIGENDUM

**DOI:** 10.1017/S0007114523000958

**Published:** 2023-11-28

**Authors:** Frans J. Kok, Martin Zarnkow, Aafje Sierksma

**Details of correction:** update email address for corresponding author.

**Existing text:** sierksma@kennisinstituutbier.nl

**Corrected text should read:** info@beerandhealth.eu

